# Levels of C-Reactive Protein and Sodium May Differentiate a Perforated Appendix from a Nonperforated Appendix in Children

**DOI:** 10.1155/2021/9957829

**Published:** 2021-06-15

**Authors:** M. Nissen, R.-B. Tröbs

**Affiliations:** ^1^Department of Pediatric Surgery, Marien Hospital, Saint Elisabeth Group, Ruhr-University of Bochum, Marienplatz 2, D-58452 Witten, Germany; ^2^Department of Pediatric Surger, Saint Johannes Hospital, Helios Group, An der Abtei 7-11, D-47166 Duisburg, Germany

## Abstract

**Background:**

Acute appendicitis (AA) might be amenable to conservative antibiotic treatment, whereas a perforated appendix (PA) necessitates surgery. We investigated the value of clinical–laboratory markers in distinguishing AA from a PA.

**Methods:**

Retrospectively obtained preoperative parameters for 306 consecutive patients (<18 years) with histologically confirmed appendicitis (AA (*n* = 237) *vs*. PA (*n* = 69)), treated at our institution between January 2014 and December 2017.

**Results:**

A PA was associated with male preponderance, younger age, decreased sodium level and increased white blood cell count, Tzanakis score, C-reactive protein (CRP) level, and CRP-to-lymphocyte ratio (CLR). Upon discrimination analysis, CLR and CRP displayed the highest accuracy in differentiating a PA from AA. Regression analysis identified levels of CRP, sodium, and the Tzanakis score as independent predictors for a PA.

**Conclusion:**

Levels of CLR, CRP, sodium, and Tzanakis score might support decision-making regarding treatment options for pediatric appendicitis.

## 1. Introduction

Appendicitis is a common general-surgical emergency. A lifetime risk of 8.6% in males and 6.7% in females, with a peak incidence during the second decade of life, has been documented for appendicitis [1, 2].

The diagnosis of appendicitis in children is challenging because of the following: (i) often atypical presentation, (ii) misleading symptomatology, (iii) preverbal status of children aged <2 years, and (iv) increased susceptibility to infectious states (e.g., mesenteric lymphadenitis) that mimic appendicitis (especially in younger children) [3]. The prevalence of misdiagnosis of acute appendicitis (AA) can reach 28–57% in children aged >2 years. [4] Moreover, children aged <1 year carry a risk of a perforated appendix (PA) of 86–100%, whereas it is ≤74% in older children with AA [2, 5]. Complications arising from a PA are associated with increased morbidity and include abscess formation, peritonitis, intestinal adhesions, ileus, chronic pain, fertility problems, prolonged duration of hospital stay (DoHS), and additional healthcare expenses [2, 6].

Advances in imaging and increased use of ultrasound (US) in children (which carries a sensitivity of 44–94% and specificity of 47–95%) have led to a decline in the prevalence of unnecessary appendectomies, but the prevalence of a PA has remained relatively unaltered [3]. Those data suggest that imaging improvements are less effective in differentiating a PA from a non-PA, which prompts the need for a diagnostic tool of more predictive value for a PA. If there is no abscess formation or pneumoperitoneum, ultrasound cannot be used to reliably differentiate between a PA and a non-PA, which necessitates surgical exploration. A laboratory parameter of sufficient predictive value for the diagnosis of appendicitis is lacking. However, the level of C-reactive protein (CRP) and white blood cell (WBC) count (including neutrophils) is considered the “gold standard” [7, 8].

Several scoring systems have been used in clinical practice [9–11]. Recently, appendicitis inflammatory score (AIR) has been created to overcome shortcomings of the most commonly used Alvarado score and Pediatric appendicitis score. Recent studies validated the AIR score and reported that the AIR score significantly outperforms the older Alvarado score, especially in distinguishing simple from advanced appendicitis but still not enough to be an exclusive criteria in establishing the diagnosis of acute appendicitis [12].

Recently, hyponatremia has been reported as a very sensitive marker of advanced acute appendicitis in children with sensitivity and specificity of 94.7% and 88.5%, respectively [13].

At our institution, surgery is undertaken on all children with appendicitis regardless of the index of suspicion for a PA. However, according to recent research, a non-PA might undergo spontaneous resolution and seems to be more receptive to conservative antibiotic treatment alone, whereas other types of appendicitis presentation with a PA before hospital admission necessitates surgery [14–16]. In this context, we wished to determine the value of specific parameters in distinguishing AA from a PA in a pediatric cohort.

## 2. Materials and Methods

### 2.1. Ethical Approval of the Study Protocol

The study protocol was approved (19-6741-BR) by the Ethics Committee of the Ruhr-University of Bochum (Germany).

### 2.2. Study Design

**Figure 1:**
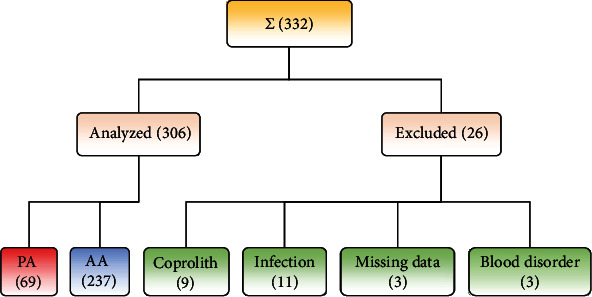
Flowchart of patients. The sample size is presented in brackets. Perforated appendicitis (PA); acute appendicitis (AA); sum of all enrolled appendicitis cases (*Σ*).

In this retrospective, single-center study, we identified 332 consecutive cases aged <18 years with the principal diagnosis of appendicitis on discharge as identified by the ICD-10-GM (International Classification of Diseases, 10^th^ Revision, German Modification) code for appendicitis (K35.) and the OPS (Operation and Procedure Coding System) code for appendectomy (5-470) during study period 01/2014–12/2017. Exclusion criteria were age above 17 years, nonavailability of laboratory (n =3) or histopathological data, and coassociated confounding factors as appendicoliths, infections (e.g., oxyuriasis and enteritis), or hematologic disorders (Figure 1). The remaining 306 cases were considered eligible for further analysis. The primary outcome of this study was the validity of several laboratory factors such as CRP, CLR, and sodium at admission for differentiation of AA from a PA. The secondary outcomes included determination of other predictive factors of PA such as age, temperature on admission, Tzanakis score, duration of the surgical procedure, and DoHS. Data were derived from clinical notes as well as surgical, laboratory, and histopathology reports.

### 2.3. Patient Grouping

Based on the histopathology classification, data were dichotomized into AA (*n* = 237) and PA (*n* = 69). As proposed by Saint Peter and colleagues, a “perforation” was defined as a hole in the appendix confirmed by histopathology [17]. As described elsewhere [18], our antibiotic regimen comprised daily intravenous administration of cefuroxime and metronidazole for coverage of anaerobic isolates. Antibiotic therapy was started at the time of the diagnosis. Surgery was done on an urgent basis when the operating theater was available. The choice of surgical technique (laparoscopic, open, or conversion from laparoscopic to open) was at the discretion of the surgeon. While open appendectomy was performed via a modified Lanz approach, laparoscopy was performed utilizing the conventional 3-port or single-site transumbilical port (LESS-A; laparoendoscopic single-site appendectomy) access to the abdominal cavity, as described in detail by Vahdad et al. [18].

Hematology parameters were obtained upon hospital admission: WBC (10^9^/L), lymphocytes (10^9^/L), CRP (mg/dL), CRP level-to-lymphocyte-count ratio (CLR; [mg/dL]/[10^9^/L]), and sodium (mmol/L). The Tzanakis scoring system was utilized for improving the predictive value of single factors in the diagnosis of appendicitis [9]. It comprises four independent clinical-laboratory variables (6 points for ultrasound demonstrating appendicitis; 4 points for tenderness in the right lower quadrant; 3 points for rebound tenderness; 2 points for WBC count >12 × 10^9^/L) with a cutoff ≥8 out of a maximum of 15 points being considered predictive of AA.

### 2.4. Data and Statistical Analyses

The sampling and analyses of data were done using Excel™ (Microsoft, Redmond, WA, USA). Statistical analyses were undertaken with OriginPro™ 2021b (OriginLab, Northampton, MA, USA) and SPSS 27 (IBM, Armonk, NY, USA) for binary regression analysis.

Data are the arithmetic mean ± SD or median and quartiles (Q1–Q3) if a nonnormal distribution was observed. Categorical variables are presented as frequencies and percentages. A normal distribution of numeric variables was confirmed by the Kolmogorov–Smirnov normality test at a significance level of 0.05. Comparisons of dichotomic variables were carried out using the Student's *t*-test for parametric data and the Mann–Whitney *U*-test for nonparametric data. The area under the receiver operating characteristic (ROC) curve of relevant parameters compared with that in their control group was calculated, and the sensitivity at 95% specificity is given. Analyses of ROC curves enabled evaluation of the optimal cutoff for variables that could be used to predict PA, and the Youden Index was used [19]. Sensitivity, specificity, positive predictive value, negative predictive value, and odds ratios (ORs) were calculated for the cutoff values selected. According to suggestions proffered by Hosmer and Lemeshow [20], variables with an area under the ROC curve (AUC) >0.7 denoted “acceptable” predictive accuracy and were considered for logistic regression analysis. *p* ≤ 0.05 was considered significant.

## 3. Results

### 3.1. Basic Data

**Figure 2:**
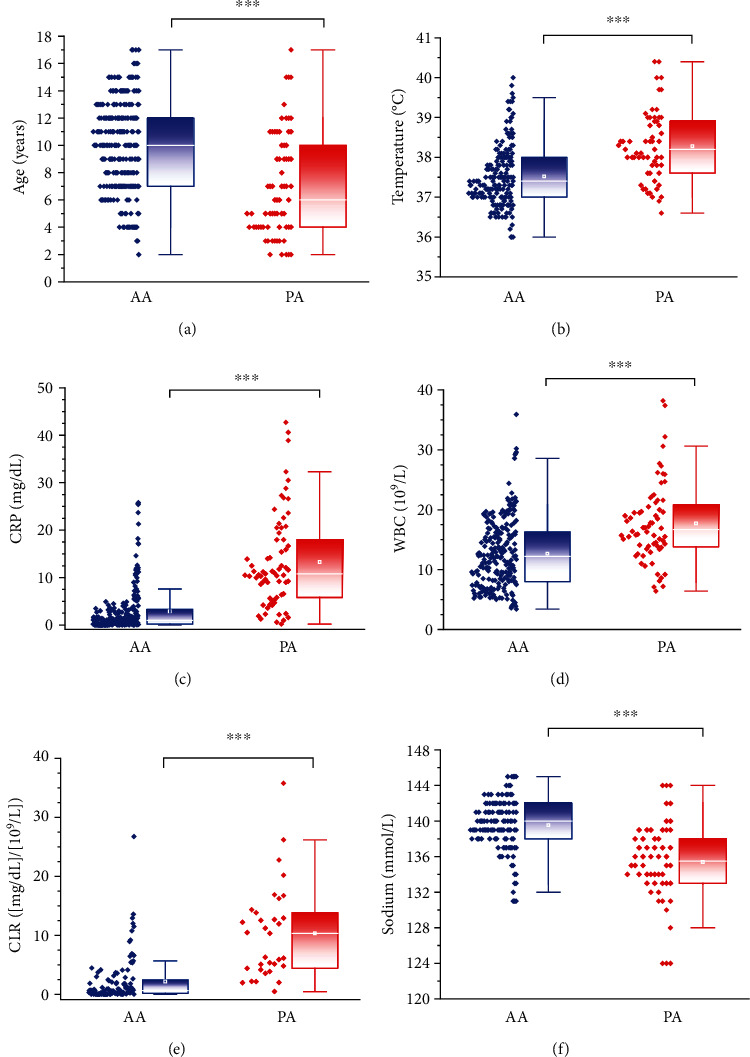
Parameters used to differentiate acute appendicitis (AA) from a perforated appendix (PA). With regard to a PA, younger age (a) and increased values for temperature (b), CRP level (c), WBC count (d), and CRP-to-lymphocyte ratio (CLR) (e) were observed, together with hyponatremia (f). Box plots and whisker plots represent median, interquartile, mean (white square and thin white line) and outlier values; raw data are depicted on the left side of each box. ^∗∗∗^ (*p* ≤ 0.001) denote significant differences.

Basic clinical, laboratory, and procedural characteristics are given in Table 1. A total of 306 patients with appendicitis confirmed by histology were evaluated. The study cohort had a male preponderance (females, *n* = 132; 43.1%). Data was dichotomized into AA (comprising catarrhal, phlegmonous, and gangrenous appendicitis; *n* = 237) and PA (*n* = 69). The overall prevalence of a PA was 22.5% (27% in males and 16.7% in females). Compared with the AA group, a PA was associated with a younger age (*p* < 0.001) (Figure 2(a)), an increased body temperature upon hospital admission (*p* < 0.001) (Figure 2(b)), and a higher Tzanakis score (*p* < 0.001). In the PA group, the CRP level (*p* < 0.001) (Figure 2(c)), WBC count (*p* < 0.001) (Figure 2(d)) and CLR (*p* < 0.001) (Figure 2(e)) upon hospital admission were increased, but the number of lymphocytes was unaltered (*p* = 0.83), compared with those in the AA group. The sodium level was reduced in the PA group (*p* < 0.001) (Figure 2(f)). Procedural parameters such as duration of the surgical procedure and DoHS were prolonged in the PA group compared with those in the AA group (*p* < 0.001 for both). The vast majority of cases (*n* = 288; 98.7% of AA cases and 78% of PA cases) were treated by conventional 3-port laparoscopic appendectomy; conversion from laparoscopy to laparotomy was necessary in 13 cases, and 5 cases had primary laparotomy.

### 3.2. ROC Characteristics

**Figure 3:**
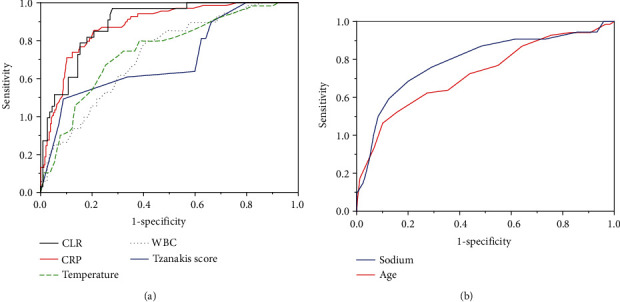
ROC curves of variables to differentiate acute appendicitis from a perforated appendix.

ROC analyses for differentiation of AA from a PA (Table 2; Figures 3(a) and 3(b)) revealed moderate diagnostic accuracy (AUC = 0.7–0.9) for the Tzanakis score, age, body temperature, CRP level, CLR, sodium level, and the WBC count.

### 3.3. Regression Analysis

The independent regression parameters were identified by AUC values ≥0.7 from ROC analysis and were considered eligible for binary logistic regression analysis (Enter method) if the variance inflation factor (VIF; as a measure of multicollinearity between variables) was within the threshold of 2.5, as proposed by Midi and colleagues [21]. Due to VIF levels of 4.011 and 3.452 for CRP and CLR, respectively, two regression models with either CRP or CLR included together with the following variables were performed: age, Tzanakis score, body temperature on admission, levels of sodium, and WBC. In brief, the CRP-controlled model slightly outperformed the CLR-controlled model. In the CRP-controlled model, among all included variables, levels of sodium (OR 0.858, 95% CI 0.737–1.000, *p* = 0.050) and CRP (1.183, 1.091–1.283, *p* < 0.001) and the Tzanakis score (1.200, 1.021–1.411, *p* = 0.027) were retained in the final model of independently associated variables with PA. However, when CRP was replaced by CLR in the regression model, the latter was the only variable independently associated with PA (1.168, 1.043-1.309, *p* = 0.007).

## 4. Discussion

We wished to evaluate the value of basic clinical–laboratory parameters in differentiating a PA from AA in a pediatric cohort. An increased CRP level and Tzanakis score, together with hyponatremia, were of independent predictive value for a PA. Moreover, in this context, the value of CLR might be equal to, or greater than, that of CRP. This information could foster decision-making towards the treatment of AA and PA cases.

The present study had several limitations. First, it was retrospective. Second, it was from a single center. Third, the study cohort was relatively small. Fourth, longitudinal laboratory data were lacking, so conclusions on possible dynamic variations in the investigated parameters could not be drawn. Fifth, the validity of data on body temperature may have been compromised by the preceding use of antipyretic medication because documentation of this issue was not part of our study protocol.

We observed reduced levels of sodium in the PA group (Table 1 and Figure 2(f)), and similar results were reported in a prospective study by Lindestam and coworkers [22]. Moreover, they found that increased concentrations of vasopressin in PA cases compared with those in patients without a PA. In this context, conditions as vomiting, pain, fever, physiological stress, and hypovolemia are often associated with a PA and may constitute nonosmotic stimuli for vasopressin release with consecutive hyponatremia [23]. The pathogenesis of hyponatremia in PA is incompletely understood, but an interleukin- (IL-) 6- and vasopressin-mediated response in early systemic inflammation might have a pivotal role [24–26]. Of note, hyponatremia (sodium <136 mmol/L) is not only associated with a PA but also with an increased morbidity and mortality [27–29]. In this context, hyponatremia has been hypothesized to have predictive value in detection of perforation in sigmoid diverticulitis in older patients undergoing emergency surgery [29] and anastomotic leakage in patients following colorectal surgery [30]. Compared to the results of a prospective study by Pogorelić et al. [13], the discriminatory accuracy of our data on sodium was lower regarding differentiation of a PA from AA. Noteworthy, cutoff was set at a lower level and also mean levels of their PA group were markedly lower. However, our finding of sodium as an independent factor using regression analysis underlines its importance as a surrogate for a PA.

In our series, the CRP level was more sensitive than the WBC count for detecting a PA (Table 2 and Figure 3(a)). Accordingly, the CRP level has been reported being more sensitive than the WBC count for detecting a PA [31]. However, these data must be interpreted with caution because each parameter follows a characteristic time course. Hence, the timepoint of sampling has considerable influence on the obtained value. Upon the onset of inflammation, the number of circulating leukocytes is increased following their decrease secondary to sequestration within inflamed tissues after ≥48 h. Serum CRP levels start to increase within 6 h from the initial insult and reach a peak at ~40 h, with a decrease thereafter [6, 31, 32].

In search of a more sensitive marker that reflects systemic inflammation and the immunological response, the CLR was applied (Tables 1 and 2, Figure 2(e) and 3(a)). To our knowledge, this is the first study to use the CLR to differentiate a PA from AA, and the first to recruit a pediatric population for its application. The hypothetical advantage of the CLR over the CRP level alone or lymphocyte number alone lies in its unified reflection of two opposing immune pathways, with a possibly more accurate consideration of temporal variations of each integrated parameter. The CLR has been proposed as an indicator of systemic inflammation in malignancies [33, 34] or intestinal ischemia [35]. With regard to appendicitis, the CLR has been applied only once, in a recent study by Daldal and Dagmura [36]. They utilized the reciprocal value, the lymphocyte-to-CRP ratio (LCR) in the context of a possibly related appendix diameter and complete blood count parameters by comparing AA with lymphoid hyperplasia (normal appendix) in adults with an appendix diameter >6 mm. However, even though the WBC count, neutrophil count, and neutrophil-to-lymphocyte ratio were altered, the LCR remained unchanged. In contrast to those results, the CLR was increased in our PA cohort (Table 1 and Figure 2(e)) and displayed among the highest discriminatory accuracy according to ROC analyses (Table 2 and Figure 3(a)). This discrepancy in results could be explained by a lower prevalence of a PA in the AA cohort in the abovementioned study by Daldal and Dagmura. Conversely, the CLR might not be applicable in adults with appendicitis. Due to the observed multicollinearity regarding the CRP level and CLR, two regression models, including either CRP or CLR, were calculated with highest accuracy in the CRP-controlled model. Perhaps longitudinal measurements instead of a single measurement might have demonstrated the superiority of the CLR over CRP in terms of a truer and more dynamic depiction of the underlying inflammatory state.

Our applied Tzanakis score served as an internal control regarding the clinical validity of laboratory results. In accordance with the literature [9], the Tzanakis score was increased in the PA group compared with that in the AA group, thereby reflecting *inter alia* clinical progression of appendiceal inflammation. Although the Tzanakis score displayed only moderate discriminatory ability (Table 2 and Figure 3(a)), together with the CRP and sodium level, it was an independent predictor for a PA in the CRP-controlled regression model.

In accordance with the literature, male sex was associated with a PA (Table 1). Also, the sex distribution in the AA group was evenly matched [37]. In line with the literature, a PA was associated with a younger age compared with AA [5]. The value of body temperature as a cofactor in predicting a PA has been described by van den Bogaard and colleagues [38]. However, apart from being higher in the PA group (Table 1), body temperature upon hospital admission was not retained in our final regression model as being independently predictive for a PA.

## 5. Conclusions

Our study provides data on the potential of using the CLR to distinguish a PA from AA in children. Also, it underlines the importance of an increased CRP level, hyponatremia, and high Tzanakis score as predictors for a PA. These factors might be helpful if clinical and imaging investigations are inconclusive. Moreover, if applicable, these factors might support decision-making towards a hypothetical conservative (antibiotic) treatment in AA or surgery to treat a PA.

## Figures and Tables

**Table 1:** Clinical, laboratory, and procedural parameters regarding acute appendicitis versus a perforated appendix.

	Acute appendicitis	*n*	Perforated appendicitis	*n*	*p*
Number	237		69		
Female : male (*n*)	110 : 127		22 : 47		
Female (%)	46		32		
Age (years)	10 (7–12)	237	6 (4–10)	69	<0.001^†^
Temperature (°C)	37.4 (37.0–38.0)	188	38.2 (37.6–38.9)	59	<0.001^†^
Tzanakis score	10 (6–12)	237	12 (9–15)	69	<0.001^†^
Laboratory data					
CRP (mg/dL)	1.0 (0.2–3.3)	236	10.8 (5.7–18.7)	69	<0.001^†^
CLR (mg/dL/10^9^/L)	0.6 (0.2–2.5)	111	10,3 (4.3–14.1)	33	<0.001^†^
Lymphocytes (10^9^/L)	1.8 ± 0.8	112	1.6 ± 0.9	33	0.26^‡^
WBC (10^9^/L)	12.7 ± 5.8	237	17.7 ± 6.6	68	<0.001^‡^
Sodium (mmol/L)	140.0 (138.0–142.0)	120	135.5 (133.0–138.3)	54	<0.001^†^
Procedural data					
DoHS (days)	4 (3–4)	237	7 (5–9)	69	<0.001^†^
Procedure duration (min)	50 (40–65)	237	80 (60–104)	69	<0.001^†^
Mode of surgery					
Laparoscopy/conversion/open (*n*)	234/1/2		54/12/3		

CRP: C-reactive protein; WBC: white blood cell. CLR: C-reactive protein-to-lymphocyte ratio; DoHS: Duration of hospital stay. ^†^Mann–Whitney *U*-test; ^‡^two-sample *t*-test.

**Table 2:** Analyses of ROC curves for selected variables.

	AUC (±SE)	Cutoff	Sensitivity	Specificity	95% CI	*p*	PPV	NPV	OR (95% CI)
Age (*y*)	0.72 ± 0.03	6,5	52%	84%	0.66–0.79	<0.001	49%	86%	5.9 (3.3–10.6)
Temperature (°C)	0.74 ± 0.04	38.0	68%	75%	0.68–0.82	<0.001	46%	88%	6.1 (3.3–11.6)
Tzanakis score	0.70 ± 0.03	12.5	49%	91%	0.63–0.77	<0.001	63%	86%	10.0 (5.2–19.2)
CRP (mg/dL)	0.88 ± 0.03	4.1	86%	79%	0.83–0.93	<0.001	55%	95%	22.5 (10.7–47.2)
CLR (mg/dL/10^9^/L)	0.89 ± 0.04	1.9	97%	72%	0.82–0.96	<0.001	42%	94%	27.0 (7.7–95.1)
WBC (10^9^/L)	0.72 ± 0.04	13.4	79%	59%	0.65–0.80	<0.001	38%	95%	12.4 (5.4–28.2)
Sodium (mmol/L)	0.79 ± 0.04	137.5	69%	80%	0.72–0.86	<0.001	61%	85%	8.7 (4.2–18.0)

AUC: area under the curve; SE: standard error; PPV: positive predictive value; NPV: negative predictive value; OR: odds ratio; CI: confidence interval; CRP: C-reactive protein; WBC: white blood cell. CLR: C-reactive protein-to-lymphocyte ratio.

## Data Availability

The data that support the findings of this study are available from the corresponding author, M.N., upon reasonable request.

## Additional Points

*Key Messages*. (i) Acute appendicitis (AA) might be amenable to conservative treatment while a perforated appendix (PA) necessitates surgery. (ii) Although distinctive and reliable markers for a PA or AA are lacking, our data indicate that a raised C-reactive protein (CRP), hyponatremia, and Tzanakis score may predict a PA. (iii) The value of the newly applied CRP-to-lymphocyte ratio might be similar to that for CRP.
